# Comparative Analysis of Predictive Biomarkers for PD-1/PD-L1 Inhibitors in Cancers: Developments and Challenges

**DOI:** 10.3390/cancers14010109

**Published:** 2021-12-27

**Authors:** Fang Yang, Jacqueline F. Wang, Yucai Wang, Baorui Liu, Julian R. Molina

**Affiliations:** 1 The Comprehensive Cancer Center of Nanjing Drum Tower Hospital, The Affiliated Hospital of Nanjing University Medical School and Clinical Cancer Institute of Nanjing University, Nanjing 210008, China; yangfangnju@hotmail.com; 2 Department of Medicine, NYU Langone Health, New York, NY 10016, USA; jacqueline.wang@nyulangone.org; 3 Division of Hematology, Mayo Clinic, Rochester, MN 55905, USA; wang.yucai@mayo.edu; 4 Division of Medical Oncology, Mayo Clinic, Rochester, MN 55905, USA

**Keywords:** immunotherapy, immune checkpoint inhibitor, PD-1, PD-L1, predictive biomarkers, tumor microenvironment, intratumor heterogeneity, adverse events

## Abstract

**Simple Summary:**

The development of immune checkpoint inhibitors (ICIs) has greatly changed the treatment landscape of multiple malignancies. However, the wide administration of ICIs is mainly obstructed by the low response rate and several life-threatening adverse events. Thus, there is an urgent need to identify sets of biomarkers to predict which patients will respond to ICIs. In this review, we discuss the recently investigated molecular and clinical determinants of ICI response, from the aspects of tumor features, clinical features, as well as tumor microenvironment.

**Abstract:**

Immune checkpoint inhibitors (ICIs) targeting programmed cell death protein 1 (PD-1)/programmed death-ligand 1 (PD-L1) have dramatically changed the landscape of cancer therapy. Both remarkable and durable responses have been observed in patients with melanoma, non-small-cell lung cancer (NSCLC), and other malignancies. However, the PD-1/PD-L1 blockade has demonstrated meaningful clinical responses and benefits in only a subset of patients. In addition, several severe and life-threatening adverse events were observed in these patients. Therefore, the identification of predictive biomarkers is urgently needed to select patients who are more likely to benefit from ICI therapy. PD-L1 expression level is the most commonly used biomarker in clinical practice for PD-1/PD-L1 inhibitors. However, negative PD-L1 expression cannot reliably exclude a response to a PD-1/PD-L1 blockade. Other factors, such as tumor microenvironment and other tumor genomic signatures, appear to impact the response to ICIs. In this review, we examine emerging data for novel biomarkers that may have a predictive value for optimizing the benefit from anti-PD-1/PD-L1 immunotherapy.

## 1. Introduction

The immune system plays an important role in the surveillance and eradication of cancer cells. However, malignant cells can take advantage of immune checkpoint pathways in order to evade the immune response. Programmed cell death protein 1 (PD-1) and its ligands, programmed death-ligand 1 (PD-L1) and PD-L2, mediate signaling processes that limit an excessive immune response and prevent autoimmunity. There is increasing evidence that PD-1 and PD-L1 expression are upregulated in multiple types of malignancy through diverse mechanisms. The increase in immune checkpoint signaling blocks cellular immunity against malignant cells. An immune-checkpoint blockade with PD-1/PD-L1 antibodies can effectively block the interactions between PD-1 and PD-L1, relieving the inhibition of cellular immunity and restoring antitumor immunity. CD8+ T cell dysfunction and exhaustion is a major barrier to current anti-cancer immunotherapies. PD-1 is a cell surface receptor that functions as a T cell checkpoint and plays a central role in regulating T cell exhaustion. ICIs targeting PD-1 can relieve exhaustion of CD8+ T cells and renew their priming, respectively, and thereby eliminate antigen-expressing cancer cells [1]. In the past few years, anti-PD-1/PD-L1 immunotherapy has demonstrated remarkable efficacy in multiple malignancies.

Immunotherapy has dramatically changed the landscape of cancer therapy due to high and durable activity in select patients with advanced cancer. However, more than half of the patients derived no benefit from an immune checkpoint blockade in most cancer types. Severe immune-related adverse events (irAEs) were associated with the administration of PD-1/PD-L1 inhibitors in some patients [2]. Hence, it is imperative to identify predictive biomarkers to help with optimal patient selection. Thus far, PD-L1 expression, tumor mutation burden (TMB), and microsatellite instability (MSI) have been used to predict the efficacy of PD-1/PD-L1 blockade. However, conflicting results surrounding the association between PD-L1 expression and clinical benefit have been demonstrated in multiple studies. Additionally, several novel tumor genomic biomarkers are emerging in the field. Features of the tumor microenvironment (TME) and patients’ clinical characteristics may also associate with the response to ICIs [3,4]. In this article, we review the current landscape of potential predictive biomarkers of anti-PD-1/PD-L1 immunotherapy.

## 2. Tumor Intrinsic-Based Biomarkers

### 2.1. PD-L1 Expression

As a target of PD-1/PD-L1 antibodies, PD-L1 expression is a logical and commonly examined biomarker for predicting the response to anti-PD-1/PD-L1 therapy. However, there is no current unified standard for defining PD-L1 positivity when analyzing the association between PD-L1 expression and the response to a PD-1/PD-L1 blockade. The definition of PD-L1 positivity varies across clinical trials, and sometimes several cutoff values were used in the same study to explore the best cutoff [5,6,7,8,9,10,11,12,13,14,15,16,17,18,19,20,21,22,23,24,25,26,27,28,29,30,31,32,33,34,35,36,37,38,39,40,41].

We found that predictive values of PD-L1 expression were inconsistent among different cancer types and different cutoffs were used to define PD-L1 positivity (Table 1). For example, the phase III clinical trial CheckMate 017 evaluated the efficacy and safety of nivolumab in advanced squamous-cell NSCLC and found that PD-L1 expression was neither prognostic, nor predictive, of any of the efficacy end points [12]. However, a subsequent trial found that a higher PD-L1 expression level was associated with superior outcomes of nivolumab in non-squamous-cell NSCLC [13]. Notably, the phase I trial CheckMate 012 found that advanced NSCLC patients could benefit from nivolumab regardless of PD-L1 expression using 1% and 5% as cutoff values, whereas nivolumab was associated with higher objective response rate (ORR) at the prespecified PD-L1 expression levels of 10% or higher, 25% or higher, and 50% or higher [10]. It is certainly possible that the immunological environment and impact of PD-L1 expression vary in different cancer types.

In theory, the predictive role of PD-L1 expression should be similar across different ICIs since they work by blocking the same pathway. However, it has recently been shown that treatment preferences varied by different PD-L1 expression thresholds. In the first-line setting of NSCLC, the most effective treatments for patients with PD-L1 expressions of ≥50%, 1–49%, and <1% were atezolizumab, pembrolizumab/chemotherapy, and nivolumab/ipilimumab, respectively [42]. Another study found that the addition of pembrolizumab or nivolumab/ipilimumab to platinum-based chemotherapy was the best therapeutic option for NSCLC with a PD-L1 tumor proportion score (TPS) of 1–49% [43].

Additional challenges also exist in defining the potential predictive role of PD-L1 expression. First, the methods and antibodies used for immunohistochemistry can introduce variations across different clinical studies. Second, it remains unclear which scoring system is most superior in determining whether to quantify PD-L1 expression with tumor cells, tumor-infiltrating immune cells, or both, as well as how to select the best cutoff values. Third, the expression of PD-L1 is likely a dynamic process. In one study, the concordance rate for PD-L1 expression classification was only 67% between paired samples collected more than 3 months apart [44]. Treatment history may also impact PD-L1 status, as some patients showed positive conversion of PD-L1 expression after chemotherapy and nivolumab treatment [45]. Furthermore, differential expression of PD-L1 between primary and metastatic sites was observed in cancers, indicating that a single biopsy may not be sufficient for the evaluation of PD-L1 expression [46,47,48]. Nonetheless, through its role as the target of PD-1/PD-L1 blockade agents, PD-L1 expression remains a promising predictive biomarker in cancer immunotherapy.

### 2.2. Microsatellite Instability (MSI)

DNA mismatch repair-deficiency (dMMR) is a cause of MSI, which plays an important role in the development of multiple cancers such as colorectal cancer (CRC) and endometrial cancer. The phase II CheckMate 142 trial demonstrated that nivolumab provided durable responses and disease control in dMMR/high-level MSI (MSI-H) metastatic colorectal cancer [22]. Based on this result, nivolumab was approved by the FDA as an optional treatment for metastatic CRC patients with dMMR/MSI-H. Of note, the frequency of MSI varies across tumors, with relatively high frequencies in endometrial (30%), colon (19%), and gastric (19%) cancers [49]. A phase II trial identified MMR status as a predictive value of clinical benefit with the use of pembrolizumab in CRC. Immune-related objective response rate (ORR) and progression-free survival (PFS) rate were both higher in dMMR CRCs compared with MMR-proficient (pMMR) CRCs [50]. This study was further expanded to 12 different tumor types to evaluate the efficacy of a PD-1 blockade. The ORR was virtually identical between CRC and other cancer subtypes (52% vs. 54%), suggesting that MMR is predictive of the response regardless of the cancer’s tissue of origin [51]. Based on this result, pembrolizumab was approved for the treatment of advanced cancers with dMMR/MSI-H. This marked one of the few times that the FDA has approved a cancer treatment based on a tissue-agnostic biomarker, or a common biomarker unrelated to tumor location or tissue origin.

There are several reasons that may explain how dMMR cancers showed better response to a checkpoint blockade. First, the increased number of mutation-associated neoantigens in dMMR tumors may stimulate antitumor immune responses, which may be further augmented with immune checkpoint inhibition by a PD-1 blockade. Second, the higher mutational load in MSI-H tumors tends to correlate with a higher prevalence of tumor-infiltrating lymphocytes (TILs) that could contribute to increased antitumor cytotoxic immune responses [52,53]. Third, it has been shown that dMMR endometrial carcinomas have a significantly increased PD-L1 expression in tumor and immune stromal cells compared to pMMR carcinomas [54]. Fourth, different signaling pathways between dMMR and pMMR tumors may lead to differences in the secretion of soluble factors that contribute to the activation of the PD-1 pathway within the TME [50,55]. Furthermore, alterations in the TGF-β signaling pathway may be another reason for the responses to anti-PD-1 therapy. TGF-β signaling plays a role in immune modulation, and the gene encoding the type II TGF-β receptor is frequently mutated in dMMR colon cancers [56].

BRCA1/2 mutations may be another potential biomarker for PD-1 blockade therapy, as it also represents DNA repair deficiency status and exhibits correlations with increased TILs and PD-L1 expression [57]. The phase I/II MEDIOLA trial has confirmed the safety and activity of durvalumab in combination with olaparib in patients with germline BRCA1/2-mutated metastatic breast cancer. However, the correlation between BRCA1/2, other DNA damage response and repair gene mutations, and response to a PD-1 blockade needs to be studied further.

**Table 1:** Summary of PD-L1 expression and associations with response rate and survival benefit of PD-1/PD-L1 inhibitors in clinical trials.

Trial Name/ID	Phase	Tumor Type	Antibody Clone	Stained Cell Types	Cutoff	ORR, % (n/N)		*p* Value ^§^	*p* Value	mPFS (95%CI) (Months)		*p* Value	mOS (95%CI) (Months)		*p* Value
						PD-L1 Positive	PD-L1 Negative			PD-L1 Positive	PD-L1 Negative		PD-L1 Positive	PD-L1 Negative	
Nivolumab															
CheckMate 017 [12]	3	NSCLC	28-8	TC	1%	17 (11/63)	17 (9/54)	0.936	-	3.3	3.1	0.698	9.3	8.7	0.556
				TC	5%	21 (9/42)	15 (11/75)	0.291	-	4.8	2.2	0.159	10.0	8.5	0.475
				TC	10%	19 (7/36)	16 (13/81)	0.641	-	3.7	2.3	0.347	11.0	8.2	0.406
CheckMate 057 [13]	3	NSCLC	28-8	TC	1%	31 (38/123)	9 (10/108)	0.002	-	4.2	2.1	0.020	17.7	10.5	0.060
				TC	5%	36 (34/95)	10 (14/136)	0.002	-	5.0	2.1	<0.001	19.4	9.8	<0.001
				TC	10%	37 (32/86)	11 (16/145)	0.002	-	5.0	2.1	<0.001	19.9	9.9	<0.001
CheckMate 063 [11]	2	NSCLC	NR	TC	5%	48 (12/25)	33 (17/51)	-	0.216	-	-	-	-	-	-
CheckMate 078 [58]	3	NSCLC	NR	TC	1%	17 (29/168)	18 (25/138)	-	0.845	2.8	2.9	-	12.0	11.4	-
CheckMate 227 ^ǂ^ [59]	3	NSCLC	28-8	TC	1%	36 (142/396)	27 (51/187)	-	0.040	5.1 (4.1–6.3)	5.1 (3.2–6.4)	-	17.1 (15.0–20.1)	17.2 (12.8–22.0)	-
CheckMate 9LA ^ǂ^ [60]	3	NSCLC	28-8	TC	1%	52 (105/203)	51 (69/135)	-	0.912	-	-		15.8 (13.8–NE)	16.8 (13.7–NE)	-
					50%	49 (37/76)	52 (137/262)	-	0.580	-	-	-	-	-	-
NCT00730639 [5]	1	NSCLC	NR	TC	5%	15 (5/33)	14 (3/35)	-	0.471	3.3 (1.8–7.5)	1.8 (1.7–2.3)	-	7.8 (5.6–21.7)	10.5 (5.2–14.8)	-
CheckMate 012 [10]	1	NSCLC	28-8	TC	1%	28 (9/32)	14 (2/14)	-	0.460	3.5 (<0.1–28.0+)	6.6 (<0.1–12.4)	-	-	-	-
				TC	5%	31 (8/26)	15 (3/20)	-	0.302	3.5 (<0.1–28.0+)	5.0 (<0.1–16.0+)	-	-	-	-
				TC	10%	40 (8/20)	12 (3/26)	-	0.038	5.2 (0.2–28.0+)	3.5 (<0.1–16.0+)	-	-	-	-
				TC	25%	44 (8/18)	11 (3/28)	-	0.014	5.8 (0.2–28.0+)	2.4 (<0.1–16.0+)	-	-	-	-
				TC	50%	50 (6/12)	15 (5/34)	-	0.014	8.3 (2.2–28.0+)	2.4 (<0.1–16.0+)	-	-	-	-
CheckMate 037 [7]	3	Melanoma	NR	TC	5%	43 (48/111)	15 (21/137)	-	<0.001	-	-	-	30.62 (20.44–NE)	9.86 (7.59–12.42)	-
CheckMate 066 [6]	3	Melanoma	NR	TC	5%	53 (39/74)	33 (45/136)	-	0.006	-	-	-	-	-	-
CheckMate 067 [8]	3	Melanoma	NR	TC	1%	54 (92/171)	35 (41/117)	-	0.002	-	-	-	NE (40.2–NE)	23.5 (13.0–36.5)	-
				TC	5%	58 (46/80)	42 (87/208)	-	0.017	-	-	-	NE (35.8–NE)	35.9 (23.1–NE)	-
				TC	10%	58 (34/59)	44 (101/229)	-	0.063	-	-	-	-	-	-
CheckMate 141 [14]	3	HNSCC	28-8	TC	1%	17 (15/88)	12 (9/73)	-	0.403	-	-	-	8.7 (5.7–9.1)	5.7 (4.4–12.7)	-
				TC	5%	22 (12/54)	11 (12/107)	-	0.064	-	-	-	8.8	7.0	-
				TC	10%	28 (12/43)	10 (12/118)	-	0.005	-	-	-	8.7	7.2	-
UMIN000005714 [15]	2	Ovarian cancer	27A2	TC	NR	13 (2/16)	25 (1/4)	0.509	-	-	-	-	-	-	-
NCT02873962 [61]	2	Ovarian cancer	28-8	TC	1%	14 (2/14)	46 (10/22)	-	0.076	-	-	-	-	-	-
				IC	5%	15 (3/20)	60 (9/15)	-	0.011	-	-	-	-	-	-
				CPS	1	18 (4/22)	57 (8/14)	-	0.029	-	-	-	-	-	-
				CPS	10	0 (0/9)	44 (12/27)	-	0.016	-	-	-	-	-	-
CheckMate 025 [17]	3	RCC	NR	TC	1%	-	-	-	-	-	-	-	21.8 (16.5–28.1)	27.4 (21.4–NE)	-
				TC	5%	-	-	-	-	-	-	-	21.9 (14.0–NE)	24.6 (21.4–NE)	-
NCT01354431 [16]	2	RCC	28-8	TC	5%	31 (9/29)	18 (14/78)	-	0.143	4.9 (1.4–7.8)	2.9 (2.1–4.2)	-	NE (13.4–NE)	18.2 (12.7–26.0)	-
CheckMate 032 [18]	1/2	UC	28-8	TC	1%	24 (6/25)	26 (11/42)	-	0.842	5.5 (1.4–11.2)	2.8 (1.4–6.5)	-	16.2 (7.6–NE)	9.9 (7.0–NE)	-
					5%	28 (4/14)	25 (13/53)	-	0.740	5.5 (1.2–11.2)	2.8 (1.5–7.0)	-	12.9 (2.8–NE)	10.4 (7.0–NE)	-
CheckMate 275 [19]	2	UC	28-8	TC	1%	24 (29/122)	16 (23/143)	-	0.116	-	-	-	11.30 (8.74–NE)	5.95 (4.30–8.08)	-
ATTRACTION-2 [20]	3	Gastric or GOJ	28-8	TC	1%	-	-	-	-	-	-	-	5.22 (2.79–9.36)	6.05 (4.83–8.54)	-
					5%	28 (23/81)	16 (29/184)	-	0.017	-	-	-	-	-	-
CheckMate 040 [21]	1/2	HCC	28-8	TC	1%	27 (3/11)	12 (4/33)	-	0.341	-	-	-	-	-	-
CheckMate 142 [22]	2	CRC	28-8	TC	1%	29 (6/21)	28 (13/47)	-	0.938	-	-	-	-	-	-
NCI-9742 [62]	2	NPC	28-8	TC	1%	39 (7/18)	14 (3/21)	-	0.141	-	-	-	-	-	-
CheckMate 358 [63]	1/2	Merkel cell carcinoma	28-8	TC	1%	71 (5/7)	47 (8/17)	-	0.386	-	-	-	-	-	-
NCT00730639 [5]	1	Advanced tumors	5H1	TC	5%	36 (9/25)	0 (0/17)	0.006	-	-	-	-	-	-	-
Pembrolizumab															
KEYNOTE-001 [23]	1	NSCLC	22C3	TC and IC	1%	27 (51/190)	10 (3/30)	-	0.065	-	-	-	-	-	-
				TC or IC	50%	42 (33/78)	15 (21/142)	-	<0.001	-	-	-	-	-	-
KEYNOTE-407 [64]	3	NSCLC	22C3	TC	1%	59 (104/176)	67 (64/95)	-	0.180	8.2 (6.3–10.2)	6.3 (6.1–8.5)	-	18.9 (14.0–22.2)	15.0 (13.2–19.4)	-
KEYNOTE-012 [24]	1b	UC	22C3	TC	1%	14 (2/14)	27 (3/11)	-	0.623	-	-	-	-	-	-
				TC or IC	1%	24 (5/21)	0 (0/4)	-	0.549	-	-	-	-	-	-
KEYNOTE-045 [26]	3	UC	22C3	TC or IC	10%	22 (16/74)	21 (41/196)	-	0.899	-	-	-	-	-	-
KEYNOTE-052 [25]	2	UC	22C3	TC or IC	1%	27 (59/219)	11 (5/46)	-	0.021	-	-	-	-	-	-
					10%	39 (31/80)	18 (33/185)	-	<0.001	-	-	-	-	-	-
KEYNOTE-012 [27]	1b	HNSCC	22C3	TC	1%	19 (17/89)	16 (7/43)	0.348	-	-	-	0.195	-	-	0.132
				TC or IC	1%	22 (23/107)	4 (1/25)	0.021	-	-	-	0.008	10.1	5.0	0.008
KEYNOTE-055 [28]	2	HNSCC	22C3	TC or IC	1%	18 (25/140)	12 (3/26)	-	0.574	-	-	-	-	-	-
				TC or IC	50%	27 (13/48)	13 (15/118)	-	0.025	-	-	-	-	-	-
KEYNOTE-012 [29]	1b	Gastric cancer	22C3	TC or IC	1%	17 (1/6)	24 (7/29)	-	1.000	-	-	-	-	-	-
				TC or IC	50%	33 (1/3)	22 (7/32)	-	0.553	-	-	-	-	-	-
KEYNOTE-059 [65]	2	Gastric or GOJ	22C3	TC or IC	1%	16 (23/148)	6 (7/109)	-	0.024	-	-	-	-	-	-
KEYNOTE-180 [66]	2	ESCC and adenocarcinoma	22C3	CPS	10	14 (8/58)	6 (4/63)	-	0.227	-	-	-	-	-	-
CP-MGAH22–05 [67]	1b/2	GOJ	22C3	CPS	1	33 (11/33)	7 (3/43)	-	0.006	-	-	-	-	-	-
KEYNOTE-086 [68]	2	TNBC	22C3	CPS	1	6 (6/105)	5 (3/64)	-	1.000	2.0 (1.9–2.1)	1.9 (1.7–2.0)	-	8.8 (7.1–11.2)	9.7 (6.2–12.6)	-
KEYNOTE-355 [69]	3	TNBC	22C3	CPS	1	-	-	-	-	7.6	6.3	-	-	-	
				CPS	10	-	-	-	-	9.7	5.8	-	-	-	-
				CPS	20	-	-	-	-	9.5	6.6	-	-	-	-
PANACEA [70]	1b/2	HER2+ BC	22C3	CPS	1	15 (7/46)	0 (0/12)	-	0.325	2.7 (2.6–4.0)	2.5 (1.4–2.7)	-	NE (13.1–NE)	7.0 (4.9–9.8)	-
KEYNOTE-100 [71]	2	Ovarian cancer	73-10	CPS	1	10 (20/197)	17 (14/82)	-	0.107	-	-	-	-	-	-
KEYNOTE-158 [72]	2	Cervical cancer	22C3	CPS	1	15 (12/82)	0 (0/15)	-	0.203	-	-	-	-	-	-
KEYNOTE-158 [73]	2	Mesothelioma	22C3	CPS	1	8 (6/77)	13 (4/31)	-	0.468	-	-	-	-	-	-
KEYNOTE-199 [74]	2	Prostate cancer	22C3	CPS	1	5 (7/133)	3 (2/66)	-	0.475	-	-	-	-	-	-
NCT02501096 [75]	2	Endometrial cancer	22C3	CPS	1	50 (6/12)	60 (6/10)	-	0.691	-	-	-	-	-	-
NCT02267603 [30]	2	Merkel cell carcinoma	22C3	TC	1%	33 (4/12)	45 (5/11)	0.610	-	-	-	-	-	-	-
CARSKIN [76]	2	CSCC	E1L3N	TC	1%	55 (22/42)	17 (2/12)	-	0.046	-	-	-	-	-	-
JVDF [77]	1a/b	Gastric or GOJ	22C3	CPS	1	24 (4/17)	8 (1/13)	-	0.355	-	-	-	-	-	-
		NSCLC	22C3	CPS	1	55 (6/11)	40 (4/10)	-	0.670	-	-	-	-	-	-
		UC	22C3	CPS	1	30 (3/10)	0 (0/10)	-	0.211	-	-	-	-	-	-
Cemiplimab															
EMPOWER-Lung 1 [78]	3	NSCLC	22C3	TC	50%	40 (85/210)	36 (26/73)	-	0.464	8.2 (6.1–8.8)	4.1 (2.6–6.1)	-	NE (17.9–NE)	16.5 (11.6–NE)	-
Sintilimab															
ORIENT-11 [79]	3	NSCLC	22C3	TC	1%	-	-	-	-	-	-	-	-	-	-
Atezolizumab															
POPLAR [35]	2	NSCLC	SP142	TC or IC	1%	18 (17/93)	8 (4/51)	-	0.137	2.8 (2.6–5.5)	1.7 (1.4–4.2)	-	15.5 (11.0–NE)	9.7 (6.7–12.0)	-
OAK [36]	3	NSCLC	SP142	TC or IC	1%	18 (43/241)	8 (14/180)	-	0.003	2.8 (2.6–4.0)	2.6 (1.7–2.9)	-	15.7 (12.6–18.0)	12.6 (9.6–15.2)	-
IMpower150 [80]	3	NSCLC	SP142	TC or IC	1%	-	-	-	-	-	-	-	24.4 (20.2–28.1)	14.8 (11.9–16.8)	-
IMpower133 [81]	3	SCLC	NR	TC or IC	1%	53 (19/36)	75 (21/28)	-	0.069	5.1 (4.2–5.6)	5.4 (4.5–5.6)	-	9.7 (7.6–17.4)	10.2 (7.9–15.7)	-
			NR	TC or IC	5%	-	-	-	-	-	-	-	21.6 (9.4–NE)	9.2 (7.6–12.2)	-
IMpassion130 [82]	3	TNBC	SP142	IC	1%	-	-	-	-	-	-	-	25.4	19.7	-
PCD4989g [83]	1	TNBC	SP142	IC	1%	12 (11/91)	0 (0/21)	-	0.121	-	-	-	10.1 (7.0–13.8)	6.0 (2.6–12.6)	-
					10%	-	-	-	-	-	-	-	12.6 (9.5–15.5)	6.7 (4.9–7.6)	-
KATE2 [84]	2	HER2+ BC	SP142	IC	1%	-	-	-	-	8.5 (5.7–NE)	6.8 (5.4–NE)	-	-	-	-
GO30140 [85]	1b	HCC	SP263	TC or IC	1%	41 (25/61)	28 (7/25)	-	0.258	5.6 (2.6–NE)	5.7 (3.6–NE)	-	-	-	-
				TC or IC	5%	46 (17/37)	31 (15/49)	-	0.145	4.1 (1.8–NE)	5.7 (3.6–NE)	-	-	-	-
				TC or IC	10%	50 (15/30)	30 (17/56)	-	0.101	3.7 (1.8–NE)	5.7 (4.4–NE)	-	-	-	-
NCT01375842 [32]	1a	RCC	SP142	IC	1%	18 (6/33)	9 (2/22)	-	0.454	5.6 (2.8–9.0)	4.5 (1.3–8.1)		NE (20.0–NE)	28.8 (16.3–28.9)	-
IMvigor010 [86]	3	UC	SP142	IC	5%	-	-	-	-	24.8 (17.2–NE)	16.4 (12.0–20.0)	-	-	-	-
NCT02108652 [33]	2	UC	SP142	IC	1%	18 (37/207)	8 (8/103)	-	0.017	-	-	-	-	6.5 (4.4–8.3)	-
				IC	5%	26 (26/100)	10 (11/107)	-	0.003	-	-	-	11.4 (9.0–NE)	-	-
NCT01375842 [31]	1a	Urothelial bladder cancer	NR	TC or IC	5%	43 (13/30)	11 (4/35)	-	0.005	-	-	-	-	-	-
Avelumab															
JAVELIN Solid Tumor [37]	1b	NSCLC	73-10	TC	1%	14 (17/122)	10 (2/20)	>0.99	-	2.8 (2.4–4.1)	1.4 (1.3–1.7)	-	8.9 (8.0–NE)	4.6 (2.8–NE)	-
				TC	5%	14 (12/84)	12 (7/58)	0.810	-	2.8 (1.6–4.3)	1.8 (1.4–2.8)	-	10.6 (7.9–NE)	8.4 (5.6–NE)	-
				TC	25%	17 (9/53)	11 (10/89)	0.450	-	2.8 (1.4–5.3)	2.5 (1.4–3.2)	-	8.44 (6.0–11.1)	8.57 (7.16–NE)	-
				IC	10%	15 (4/27)	13 (15/115)	0.760	-	2.0 (1.3–3.5)	2.6 (1.6–3.6)	-	8.5 (3.9–NE)	8.9 (7.9–NE)	-
JAVELIN Lung 200 [87]	3	NSCLC	73-10	TC	50%	-	-	-	-	-	-	-	13·6 (10·1–18·5)	9·4 (7·5–10·8)	-
				TC	80%	-	-	-	-	-	-	-	17·1 (10·6–25·0)	9·4 (7·9–10·7)	-
JAVELIN Solid Tumor [38]	1b	BC	73-10	TC	1%	2 (2/85)	4 (2/51)	0.631	-	1.38 (1.33–1.40)	1.40 (1.38–1.40)	-	6.5 (2.7–9.2)	8.3 (6.3–NE)	-
				TC	5%	4 (1/23)	3 (3/113)	0.528	-	1.40 (1.33–1.66)	1.38 (1.38–1.40)	-	6.5 (2.2–NE)	7.5 (5.1–11.3)	-
				TC	25%	0 (0/3)	3.0 (4/133)	>0.99	-	1.40 (1.26–NE)	1.38 (1.38–1.40)	-	9.2 (NE–NE)	6.8 (4.9–10.8)	-
				IC	10%	17 (2/12)	2 (2/124)	0.039	-	1.42 (0.54–5.62)	1.38 (1.38–1.40)	-	11.3 (1.4–NE)	6.8 (4.7–9.2)	-
JAVELIN Solid Tumor [88]	1b	UC	73-10	TC	5%	24 (15/63)	13 (10/76)	-	0.104	11.9 (6.1–18.0)	6.1 (5.9–8.0)	-	8.2 (5.7–13.7)	6.2 (4.3–14.0)	
JAVELIN Bladder 100 [89]	3	UC	SP263	TC or IC	25%	-	-	-	-	5.7 (3.7–7.4)	3.0 (2.0–3.7)	-	NE (20.3–NE)	18.8 (13.3–22.5)	
NCT01772004 [39]	1b	UC	73-10	TC	1%	50 (7/14)	4 (1/23)	0.002	-	11.2 (2.6–NE)	1.7 (1.3–2.8)	-	NE (8.5–NE)	14.0 (2.7–NE)	-
				TC	5%	54 (7/13)	4 (1/24)	0.001	-	11.2 (2.6–NE)	1.7 (1.3–2.8)	-	NE (8.5–NE)	12.9 (2.7–NE)	-
				TC	25%	20 (1/5)	22 (7/32)	>0.99	-	2.8 (1.2–11.2)	2.8 (1.4–5.5)	-	NE (8.5–NE)	14.3 (4.4–NE)	-
				IC	10%	50 (1/2)	20 (7/35)	0.390	-	NE (2.7–NE)	2.8 (1.4–4.1)	-	NE (NE–NE)	14.0 (8.5–NE)	-
JAVELIN Renal 101 [90]	3	RCC	SP263	IC	1%	55 (149/270)	47 (62/132)	-	0.121	-	-	-	-	-	-
JAVELIN Merkel 200 [91]	2	Merkel cell carcinoma	73-10	TC	1%	36 (21/58)	18 (3/16)	-	0.238	-	-	-	-	-	-
				TC	5%	58 (11/19)	24 (13/55)	-	0.006	-	-	-	-	-	-
NCT02155647 [40]	2	Merkel cell carcinoma	73-10	TC	1%	34 (20/58)	19 (3/16)	-	0.361	-	-	-	-	-	-
Durvalumab															
MYSTIC [92]	3	NSCLC	SP263	TC	1%	-	-	-	-	-	-	-	14.6 (10.5–17.7)	10.1 (6.7–12.2)	-
PACIFIC [93]	3	NSCLC	SP263	TC	1%	-	-	-	-	17.8 (16.9–NE)	10.7 (7.3–NE)	-	NE (43.3–NE)	33.1 (20.8–NE)	-
					25%	-	-	-	-	17.8 (11.1–NE)	16.9 (11.0–NE)	-	NE (NE–NE)	39.7 (33.1–NE)	-
SAFIR02-BREAST IMMUNO [94]	2	TNBC	SP142	IC	1%	-	-	-	-	-	-	-	27.3 (10.5–NE)	19.5 (5.0–29.4)	-
MEDIOLA [95]	1/2	BC	SP263	TC	1%	80 (8/10)	53 (9/17)	-	0.230	-	-	-	-	-	-
			SP263	IC	1%	65 (11/17)	60 (6/10)	-	1.000	-	-	-	-	-	-
NCT01693562 [41]	1/2	UC	SP263	TC or IC	25%	28 (27/98)	5 (4/79)	-	<0.001	2.1 (1.4–2.8)	1.4 (1.3–1.5)	-	20.0 (11.6–NE)	8.1 (3.1–NE)	-
MEDI4736 [96]	1/2	Urothelial bladder cancer	SP263	TC	25%	47 (7/15)	22 (6/27)	-	0.163	-	-	-	-	-	-
			SP263	IC	25%	56 (10/18)	13 (3/24)	-	0.006	-	-	-	-	-	-
			SP263	TC or IC	25%	46 (13/28)	0 (0/14)	-	0.002	-	-	-	-	-	-
NIBIT-MESO-1 ^φ^ [97]	2	Mesothelioma	SP263	TC	0%	30 (7/23)	27 (4/15)	0.800	-	8.5 (7.8–9.0)	5.2 (4.5–5.8)	0.380	-	-	-
			SP263	TC	1%	35 (7/20)	22 (4/18)	0.390	-	11.7 (6.9–16.5)	5.2 (4.5–5.8)	0.130	-	-	-
			SP263	TC	5%	35 (6/17)	24 (5/21)	0.440	-	8.5 (7.7–9.1)	5.7 (4.9–6.4)	0.510	-	-	-
			SP263	TC	10%	27 (3/11)	30 (8/27)	0.880	-	8.5 (7.5–9.4)	5.7 (2.9–8.1)	0.750	-	-	-
			SP263	TC	25%	43 (3/7)	26 (8/31)	0.370	-	8.5 (8.2–8.7)	5.2 (2.8–7.3)	0.870	-	-	-
			SP263	TC	50%	25 (1/4)	29 (10/34)	0.850	-	11.7 (8.9–14.5)	5.2 (2.4–7.9)	0.990	-	-	-

ORR: objective response rate; OS: overall survival; PFS: progression-free survival; PD-L1: programmed death-ligand 1; TC: tumor cell; IC: immune cell; NSCLC: non-small-cell lung cancer; RCC: renal cell carcinoma; UC: urothelial carcinoma; GOJ: gastro-oesophageal junction cancer; ESCC: esophageal squamous cell cancer; HCC: hepatocellular carcinoma; TNBC: triple-negative breast cancer; BC: breast cancer; HNSCC: head and neck squamous cell carcinoma; NR: not reported; CSCC: cutaneous squamous cell carcinoma; HER2: human epidermal growth factor receptor 2; CPS: combined positive score; NE: not estimable. ^ǂ^ Combined nivolumab with ipilimumab. ^φ^ Combined durvalumab with tremelimumab. ^§^ Reported by original articles. Calculated by Chi-square test or Fisher’s.

### 2.3. Tumor Mutation Burden (TMB)

Advanced sequencing techniques have enabled a better characterization of the mutational landscape to identify patients who are more likely to derive clinical benefit from immunotherapies. It was reported that higher nonsynonymous TMB correlated with improved ORR, durable clinical benefit, and PFS in NSCLC patients treated with pembrolizumab (Table 2) [98]. High TMB was associated with improved survival in patients receiving ICIs across various cancer types. Additionally, other factors associated with mutation burden, such as smoking signature, higher neoantigen burden, and DNA repair pathway mutations, were also correlated with treatment efficacy [98]. This supports the concept that the mutational landscape may determine the sensitivity to a checkpoint blockade. The influence of an increased mutation burden on tumor immunogenicity may be explained by the role of neoantigens, which are due to somatic mutations and important in immune responses [98].

However, multiple challenges remain in the application of TMB as a reliable biomarker to predict the response to immunotherapy. First, TMB assessment is not standardized across research and clinical studies due to different detecting technologies, platforms, and calculation methods [99]. Second, a single and fixed TMB threshold that is applicable across different tumors may be difficult to define, as the number of somatic mutations differs across tumor types [99,100,101]. Third, the generation of reliable TMB results relies on strict technical specifications, as well as other factors including assay turnaround time, runtime, and cost [99]. Moreover, there have been conflicting reports on whether high TMB is associated with response to PD-1/PD-L1 inhibitors. A recent analysis failed to support the application of high TMB as a biomarker for treatment with ICIs in all solid cancer types [102]. Thus, populations with high TMB should be further classified to improve its efficacy of prediction.

**Table 2:** Summary of potential predictive biomarkers in the treatment of PD-L1/PD-1 inhibitors.

Predictive Biomarkers	Assays	Results or Conclusions
Tumor intrinsic-based biomarkers		
PD-L1 [13,35,103]	IHC	PD-L1 expression correlated with improved survival or responses to PD-1/PD-L1 inhibitors.
CTLA4, TH1, absence of CX3CL1 [103]	PCR	CTLA4, TH1, and absence of CX3CL1 correlated with responses to the anti-PD-L1 antibody.
MMR-deficiency/MSI-H [104,105]	IHC	MMR-deficinecy correlated with improved survival or responses to PD-1 blockade.
TMB [98,106]	NGS	Higher nonsynonymous TMB correlated with improved response, durable clinical benefit, and PFS.
Neoantigen burden [107]	WES	Higher neoantigen burden correlated with improved theraputic efficacy.
IFN-γ-related gene signatures [19,35,108]	Gene expression profile	Higher IFN-γ signatures correlated with response and survival benefit.
EGFR mutation [105,109]	DNA sequencing	Patients with wild-type EGFR derived survival benefit from PD-1/PD-L1 blockades whereas those with EGFR mutation did not.
KRAS mutation [13]	DNA sequencing	Patients with KRAS mutation derived survival benefit from PD-1/PD-L1 blockades whereas those with wild-type KRAS did not.
JAK1/2 mutations, B2M mutation [110,111]	WES	JAK1/2 and B2M mutations correlated with acquired resistance to PD-1 blockade.
PTEN loss [112]	WES	PTEN Loss correlated with resistance to anti-PD-1 therapy.
POLE mutation [113,114]	NGS	POLE mutation correlated with high mutational burden and elevated expression of immune checkpoint genes.
EPIMMUNE-positive/unmethylated status of FOXP1 [115]	Infnium MethylationEPIC Array	EPIMMUNE-positive signature and unmethylated status of FOXP1 correlated with improved PFS and OS with PD-1 blockers.
ITH [116]	WES	High ITH was more likely to have disease progression during immunotherapy.
Tumor microenvironment-based biomarkers		
PD-L1 in ICs [33]	IHC	High PD-L1 expression on ICs correlated with improved response.
TILs (CD8+ T cells) [117]	IHC	Baseline or post-treatment increase in TILs correlated with responses.
Early TILs and macrophages increase [118]	IHC	Increased TILs and macrophages from baseline correlated with response to anti-PD-1 antibody treatment.
HLA-I LOH [119]	DNA sequencing/HLA typing assay	HLA-I LOH correlated with poor response and survival benefit.
INF-γ, IL-6, IL-10 [120]	Human Cytokine Antibody Array	Baseline INF-γ, IL-6, and IL-10 were higher in the responders than non-responders.
REC, RLC [121]	Complete blood counts test	Baseline high REC and RLC correlated with favorable OS of patients treated with PD-1 inhibitors.
NLR, PLR [122,123,124]	Complete blood counts test	Baseline high NLR and PLR correlated with poor survival and lower responses of patients treated with PD-1 inhibitors.
Early IL-8 decrease [125]	ELISA	Early decrease of IL-8 level correlated with responses to PD-1 inhibitors.
TNF-α decrease [126]	Multiplex Assay System	TNF-α decrease correlated with responses to nivolumab.
Early Th9 cell increase [127]	Flow cytometry	Early increase in Th9 cells correlated with improved responses to nivolumab.
Early ALC increase, early ANC decrease [128]	Complete blood counts test	Early ALC increase and early ANC decrease correlated with favorable OS of patients treated with nivolumab.
Patient-based biomarkers		
Sex [78]	-	Males derived survival benefit from PD-1/PD-L1 blockades whereas females did not.
Age [129]	-	Patients <65 years old derived survival benefit from PD-1/PD-L1 blockades whereas those ≥65 did not.
ECOG PS [92]	-	ECOG PS ≥1 or ≥2 correlated with poor OS or PFS in patients treated with nivolumab.
Current or former smoker [36]	-	Current or former smokers derived survival benefit from PD-1/PD-L1 blockades whereas the patients who never smoked did not.
Tumor size [128]	-	Maximum tumor diameters of ≥30 mm correlated with poor OS in patients treated with nivolumab.
No baseline metastases (brain, liver) [26,79]	-	Patients without baseline metastasis derived survival benefit from PD-1/PD-L1 blockades whereas those with metastasis did not.
Gut microbiome [130,131,132]	WGS/16s rRNA sequencing	Gut microbiome correlated with responses to immune checkpoint blockade.
Antibiotics use [133,134]	-	Antibiotics use correlated with poor PFS and OS in patients receving PD-1 blockers.
Corticosteroid use [135]	-	High-does corticosteroid use correlated with inferior clinical benefit.
Viral infection (EBV, HPV, MCPyV) [30,136,137]	-	Viral infection correlated with increased immune cytolytic activity and high response.
Previous radiotherapy [138]	-	Previous radiotherapy correlated with longer PFS and OS with pembrolizumab treatment.
Response to the treatment immediately before nivolumab monotherapy [139]	-	Response to the treatment immediately before nivolumab monotherapy correlated with responses.
A long-time lapse from diagnosis to anti-PD-1 initiation [140]	-	Median time from diagnosis to anti-PD-1 initiation was longer among responders than non-responders.
irAEs (rash, vitiligo, colitis, etc.) [141,142,143,144]	-	High occurrence of irAEs correlated with improved survival and responses to PD-1 blockades.

PD-1: programmed cell death protein 1; PD-L1: programmed death-ligand 1; ICs: immune cells; TILs: tumor-infiltrating lymphocytes; MMR: mismatch-repair; MSI-H: high level microsatellite instability; TMB: tumor mutation load; bTMB: tumor mutation load in blood; EPIMMUNE: epigenomic profile based on a microarray DNA methylation signature; LDH: lactate dehydrogenase; CRP: C-reactive protein; REC: relative eosinophil count; RLC: relative lymphocyte count; PLR: platelet-to-lymphocyte ratio; NLR: neutrophil-to-lymphocyte ratio; ALC: absolute lymphocyte count; ANC: absolute neutrophil count; ITH: intratumor heterogeneity; ECOG PS: Eastern Cooperative Oncology Group Performance Status; EBV: Epstein–Barr virus; HPV: human papillomavirus; MCPyV: Merkel cell polyomavirus; irAEs: immune-related adverse events; NSCLC: non-small-cell lung cancer; UC: urothelial carcinoma; RCC: renal cell carcinoma; PFS, progression-free survival; OS, overall survival; immunohistochemistry: IHC; PCR: polymerase chain reaction; NGS: next generation sequencing; WES: whole-exome sequencing; EGS: whole-genome sequencing; ELISA: enzyme linked immunosorbent assay.

### 2.4. Drive Mutation Status

In addition to the total mutation load, some driver mutations may have an impact on immunotherapy efficacy. In the phase III CheckMate 057 study, NSCLC patients with wild-type EGFR or a KRAS mutation derived a significant OS benefit from nivolumab, whereas those with an EGFR mutation or wild-type KRAS did not [13]. Similar results were achieved in the following KEYNOTE-010 [145], OAK [36], and PACIFIC trials [146], which showed that previously treated NSCLC patients with wild-type EGFR derived a survival benefit from ICIs, but those with an EGFR mutation did not. However, accumulating evidence suggests that EGFR-mutant NSCLC patients may also benefit from immunotherapy. One reason might be that different subtypes of EGFR mutations may influence the response to immunotherapy. The EGFR exon 20 mutation was associated with better outcomes from ICIs compared to EGFR-sensitizing mutations [147]. A retrospective single-center study found that NSCLC patients harboring L858 mutations had a similar benefit from ICI treatment compared to those with wild-type EGFR [148]. Patients without T790M mutations or with uncommon EGFR mutations were more likely to benefit from ICIs among those with EGFR-mutated NSCLC [149,150]. Another reason for the benefit seen in EGFR-mutant NSCLC patients could be the co-expression status of other potential biomarkers. The phase II ATLANTIC trial assessed durvalumab as a third-line or later treatment for advanced NSCLC patients. Trends toward a better response and overall survival (OS) were still observed in PD-L1 TPS ≥ 25% EGFR+/ALK+ patients compared with PD-L1 TPS < 25% EGFR+/ALK+ patients [151]. Furthermore, EGFR-mutant NSCLC patients can significantly benefit from a combination therapy of immunotherapy and chemotherapy when targeted therapy has failed [152]. Therefore, patients with EGFR mutations might still benefit from anti-PD-1/PD-L1 antibodies when given in combination with chemotherapy or if there is high PD-L1 expression.

Some other loss-of-function mutations correlate with a lack of response to a PD-1 blockade. Loss-of function mutations of JAK1/2 or PTEN can lead to both an acquired and primary resistance to anti-PD-1 therapy in melanoma and metastatic uterine leiomyosarcoma [110,111,112]. A microarray DNA methylation signature (EPIMMUNE)-positive signature and unmethylated status of FOXP1 were associated with improved PFS and OS in NSCLC patients treated with PD-1 blockers [115]. Thus, co-occurring genomic alterations have emerged as potential biomarkers for immunotherapy through their impact on the tumor microenvironment [153]. KRAS-mutant tumors harboring co-mutations in TP53 exhibit a better response to anti-PD-1/PD-L1 therapy. Further studies have demonstrated that TP53 co-mutations were correlated with an inflamed tumor immune microenvironment and higher tumoral PD-L1 expression in KRAS-mutant cancers [153]. However, the evidence of co-mutations in immunotherapy is still limited, and the mechanisms that influence the immune microenvironment remain incompletely understood.

### 2.5. Tumor Neoantigen Burden (TNB)

Neoantigens are mutations encoding immunologically active proteins, which can promote the immune system to recognize the affected cell as foreign. A high TNB was associated with an improved response to ICIs in melanoma and NSCLC, likely due to the potential ability of clonal neoantigens to promote the priming and infiltration by neoantigen reactive-T cells expressing high levels of PD-1 [98,107,154]. A fitness model for tumors based on the immune interactions of neoantigens has been established to predict the response to immunotherapy [155]. Neoantigens show great potential in personalized immunotherapies, which can be designed to be the target of vaccines or cellular therapy products [156].

Despite these promising findings, TNB is facing many challenges in achieving a precise prediction of response to immunotherapy. It is expensive and time-consuming to identify neoantigens and transfer them into production [156]. Tumor heterogeneity could be an additional obstacle that reduces the accuracy of prediction. Cytotoxic chemotherapy-induced subclonal neoantigens were enriched in certain poor responders to an immune checkpoint blockade. Patients with no durable benefit of a PD-1 blockade exhibited a significantly higher neoantigen intratumor heterogeneity than patients with a durable clinical benefit [107].

### 2.6. Intratumor Heterogeneity (ITH)

Intratumor heterogeneity (ITH) has been extensively documented in various cancers and poses a substantial challenge in the development of personalized or precision cancer medicine. In previous studies conducted by our group, ITH was determined to be a potential prognostic factor of early recurrence or metastasis in triple-negative breast cancer [157,158]. Recently, the association between ITH and a response to immunotherapy has also been explored. Patients with high ITH (>50% subclonal mutations) were substantially more likely to have disease progression than a complete or partial response across all tumors [116]. The impact of ITH on the immune response was explored in another study through syngeneic mouse models of melanoma and human patient data. These models demonstrated that lower ITH predicted better immunotherapy outcomes more accurately than did mutational burden [159]. Further efforts are required to quantify the effect of ITH on an immunotherapy response with large prospective clinical studies.

## 3. Tumor Microenvironment (TME)-Based Biomarkers

**Figure 1:**
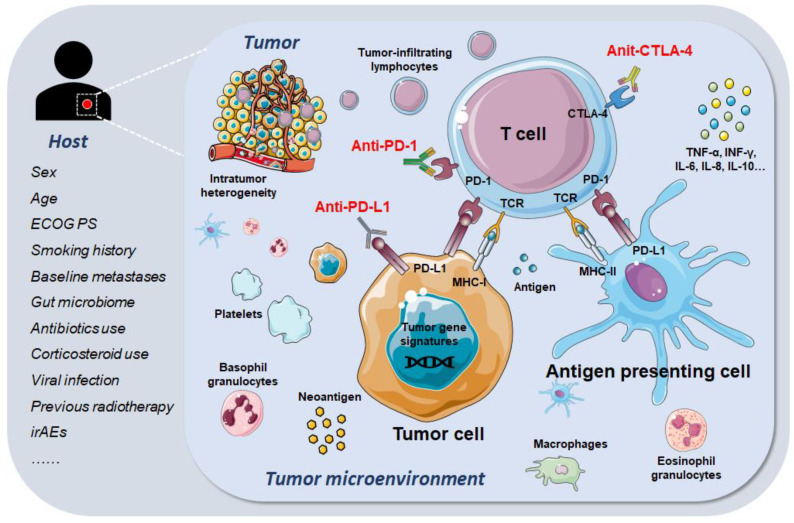
Host–tumor interaction and tumor microenvironment, along with potential biomarkers for immunotherapy. ECOG PS, Eastern Cooperative Oncology Group performance status; irAEs, immune-related adverse events; PD-1, programmed cell death protein 1; PD-L1, programmed death-ligand 1; CTLA-4, cytotoxic T lymphocyte-associated antigen-4; TCR, T cell receptor; MHC-I, Major histocompatibility complex class I; MHC-II, Major histocompatibility complex class II.

The TME has recently emerged as a key component in immunotherapy. The TME consists of a heterogenous milieu that can influence the behavior of immune cells, stromal cells, the extracellular matrix, vasculature, and cytokines. Since both immunocytes and cytokines play a critical role in mediating immune surveillance and regulating tumor growth, the heterogeneous composition of the TME might influence the efficacy of immunotherapy (Figure 1).

### 3.1. PD-L1

Currently, there are no well-established criteria for PD-L1-scoring in malignancies. One of the most-used scoring systems is the TPS, which considers the percentage of tumor cells demonstrating membrane PD-L1 staining. As previously described, patients with a higher expression of PD-L1 in tumor cells are more likely to respond to anti-PD-1/PD-L1 antibodies compared to those with lower PD-L1 expression. A superior correlation with immunotherapy response was observed when using the combined positive score (CPS), which considers the proportion of PD-L1 staining in both tumor and immune cells [160,161]. It has shown that PD-L1 expression in antigen-presenting cells, rather than in tumor cells, plays an essential role in PD-1/PD-L1 blockade therapy [162]. The expression of PD-L1 on macrophages and dendritic cells in cancer patients correlated with the efficacy of anti-PD-1 treatment [163]. PD-L1 signaling in antigen-presenting cells negatively regulated and inhibited T cell activation. Thus, PD-L1 expression in immune cells also plays a crucial role in checkpoint blockade therapy [162]. However, another retrospective study found that the TPS and CPS scoring methods were equal in predicting the response to anti-PD-1/PD-L1 therapy [164]. It remains unclear which scoring system is best for PD-L1 assessment. Moreover, there is no consensus in CPS scoring across different cancer types (Table 1). In patients with gastro-oesophageal adenocarcinoma, positive PD-L1 (CPS ≥ 1) was associated with a better response rate to pembrolizumab compared with negative PD-L1 (CPS < 1) [67]. However, in triple-negative breast cancer, the ORRs of pembrolizumab were similar in PD-L1-positive (CPS ≥ 1) and PD-L1-negative (CPS < 1) populations [68].

### 3.2. Tumor-Infiltrating Lymphocytes (TILs)

Immune recognition of tumor cells can lead to a host-immune response or T-cell-inflamed tumor phenotype to improve disease control [165]. The high density of CD8+ cells in tumor sites is suggestive of a specific immune response to tumor antigens [117]. Therefore, TILs may be able to serve as a predictive biomarker in immune checkpoint blockade therapy. Recent studies have shown that lymphocyte infiltration correlates with improved clinical outcomes in multiple cancers [166,167,168]. The predictive role of TILs in immunotherapy was first evaluated in a phase II study of ipilimumab in metastatic melanoma patients [169]. Although there was no convincing evidence that baseline TILs correlated with clinical activity, a statistically significant association was observed between changes in TILs from baseline and the clinical activity of nivolumab. The role of CD8+ and CD4+ TILs as predictive factors for anti-PD-1 therapy was evaluated in patients with NSCLC and melanoma, and an association was found between CD8+/CD4+ TIL ratios and an increased response in both cancers [170].

It remains unclear whether TILs can be a reliable predictor for response to ICIs in clinical practice. No validated predictive function of CD8+ T-cells was observed in a study of atezolizumab in cancer patients. Non-functional immune responses may explain why the pre-treatment of CD8+ T-cells in tumors failed to predict responses to atezolizumab [103]. Interestingly, in the same study, a response was observed in patients with higher levels of PD-L1, particularly when PD-L1 was expressed by TILs [103]. This suggests that the level of PD-L1 expression combined with TIL density may serve as a predictive biomarker in cancer immunotherapy. It is yet to be addressed whether other genetic signatures or immune markers can be combined with TIL quantity to predict the response to a PD-1/PD-L1 blockade. A simple measurement of TIL and clinically meaningful thresholds of increased TIL density need to be confirmed by more studies. Fortunately, a global group of breast pathologists has established a standardized and reproducible method to quantify the proportion of TILs using a microscope or a digital whole-slide image of HE staining [171].

### 3.3. Human Leukocyte Antigen (HLA)

HLAs, also known as major histocompatibility complexes, are encoded by a highly polymorphic set of genes including HLA class I (HLA-I) and class II (HLA-II) molecules. HLA molecules are expressed on the surfaces of different immune cells and play a crucial role in antigen presentation and immune signal transduction. The disruption of HLA-I antigen processing and presentation machinery mediate immune evasion and serve as a mechanism of acquired resistance to ICIs in cancer [172,173]. The loss of heterozygosity at the HLA-I locus (HLA-I LOH) has recently been described as a frequent phenomenon in cancer. The prevalence of HLA-I LOH varied widely across tumor types, ranging from 2% to 42%, with the highest rate in squamous cell cancers (30%), followed by non-squamous carcinomas (16%) and neuroendocrine tumors (11%) [174]. HLA-I LOH was determined as a putative mechanism of evading immune surveillance and was associated with the upregulation of cytolytic activity, PD-L1 positivity, and a high subclonal neoantigen burden in NSCLC [175]. Further studies demonstrated that the HLA-I genotype influenced the response to ICIs. Patients with the HLA-B44 supertype had a better survival benefit of checkpoint blockade immunotherapy, whereas the HLA-B62 supertype or somatic HLA-I LOH was associated with poorer outcomes [119].

Although HLA genotype has shown promising results, PD-L1 expression, TMB, and cancer gene mutations were found to be stronger in predicting the benefit from an immune checkpoint blockade [176]. A combination of HLA genotype along with other biomarkers might be a valuable method to enhance predictive ability. Combining HLA-I LOH with TMB significantly has been shown to improve survival prediction of benefit from ICIs [174]. HLA-corrected TMB demonstrated a predictive and prognostic value for immunotherapies and may reconcile the observed disparity in the association between TMB and responses to a PD-1/PD-L1 blockade [177]. Additional investigation is warranted to further explore the role of HLA LOH in immunotherapy.

### 3.4. Serum Cytokines and Biochemical Indicators

Due to the relative difficulty in obtaining tumor tissue samples, blood-based biomarkers remain an attractive option. Cytokines allow for immune activation and chemokines attract CD8+ T cells, both of which are prerequisites for an effective anti-PD-1/PD-L1 therapy [178]. A phase II trial of nivolumab in advanced melanoma patients assessed serum levels of immune modulators at multiple time points. It showed that the pretreatment serum IFN-γ, interleukin (IL)-6, IL-8, and IL-10 levels were significantly higher in the responding group than the disease-progressing group, suggesting a possible role of pretreatment serum cytokines in predicting immunotherapy efficacy [120,125].

IFN-γ–related gene expression signatures may also be markers of immunity against cancer and may predict a benefit from immunotherapy. It has been reported that IFN-γ–related gene expression signatures constructed with 10 genes or 28 genes are able to predict the response to pembrolizumab in patients with metastatic melanoma [179]. In the CheckMate 275 and KEYNOTE-028 trials, higher scores of the 25-gene or 6-gene IFN-γ signature were demonstrated to be associated with a better immunotherapy response in urothelial and esophageal carcinomas [19,180]. The exploratory analyses from the POPLAR trial revealed that high T-effector-IFN-γ–associated gene expression predicted an OS benefit from atezolizumab over docetaxel, whereas in patients with low T-effector-IFN-γ–associated gene expression, there was no significant difference in OS between those treated with atezolizumab or docetaxel [35].

### 3.5. Blood Cell Count

Peripheral white blood cell counts (eosinophil, lymphocyte, neutrophil, etc.) can reflect inflammatory status, and their role of predicting immunotherapy efficacy in malignancies is a growing area of research. High relative eosinophil counts (≥1.5%) and relative lymphocyte counts (≥17.5%) were demonstrated to be independent baseline characteristics that were correlated with longer OS in melanoma patients treated with pembrolizumab [121]. A new score, termed the risk blood biomarker, is calculated by combining the white blood cell count and neutrophil-monocyte-to-lymphocyte ratio, and was associated with a better survival and response to immunotherapy [180]. Elevated pretreatment neutrophil-to-lymphocyte ratio and platelet-to-lymphocyte ratio were independently associated with poorer OS and PFS and lower response rates in NSCLC patients treated with nivolumab [122]. In stage III-IV melanoma patients treated with nivolumab, high absolute lymphocyte count (≥1000/μL) and low absolute neutrophil count (<4000/μL) early during therapy (3 weeks and 6 weeks) had a significant association with a better OS [128]. While these studies seem to consistently show a predictive role of relative lymphocyte count in patients treated with checkpoint inhibitors, further prospective evaluations are needed.

## 4. Patient-Based Biomarkers

### 4.1. General Clinical Characteristics

Subgroup analyses in clinical trials revealed that younger [129], male [78], and better Eastern Cooperative Oncology Group performance status (ECOG PS) [92] patients were more likely to achieve remarkable prolonged survival with anti-PD-1/PD-L1 therapies versus chemotherapy or placebo. However, we have demonstrated that the response to ICIs does not appear to differ with regards to patient age, sex, and ECOG PS [181]. Smokers were more likely to achieve remarkable prolonged survival with anti-PD-1/PD-L1 therapies than non-smokers [60]. Additionally, NSCLC patients without baseline brain or liver metastases significantly benefit from anti-PD-1/PD-L1 therapies, whereas no differences existed for those patients with baseline brain or liver metastases [79,182].

A multiple-factor-based scale was developed to evaluate the response to immunotherapy in patients with melanoma [183]. The prediction scale was composed of five factors and in need of further validation: elevated LDH, age < 65 years, female sex, history of ipilimumab treatment, and liver metastases. While some clinical characteristics may have roles as predictive markers, excluding confounding factors can be difficult. Whether clinical characteristics can be reliable predictive markers is unclear.

### 4.2. Gut Microbiome and Antibiotics (ATB) Use

Multiple studies have reported the association between gut microbiota and its effects on immunotherapy [131,132,184,185]. Studies have determined that *Akkermansia muciniphila* and *Ruminococcaceae* bacteria were more frequently detectable in responders than non-responders to immune checkpoint blockade [131,132]. Other bacteria, including *Bacteroides stercoris*, *Parabacteroides distasonis*, and *Fournierella massiliensis* were found to be enriched in responders compared with non-responders of a combined immune checkpoint blockade targeting both CTLA-4 and PD-1 [130]. One study recently observed that bacteria expressing SagA improve the efficacy of checkpoint inhibitor therapy in animal models [186]. In addition, a fecal microbiota transplant has been proven to promote the response to anti–PD-1 therapy by reprogramming the tumor microenvironment in immunotherapy-refractory melanoma patients [187,188].

A retrospective analysis assessed the impact of ATB use on OS and PFS in subsequent-line treatment of NSCLC. The results showed that OS was significantly shortened in patients receiving ATB in the atezolizumab group, but there was no correlation between the use of ATB and survival in the docetaxel group [134]. In contrast, prior ATB therapy did not impair survival in first-line chemo-immunotherapy of NSCLC patients [133]. A meta-analysis indicated that ATB exposure shortly before or after ICI initiation led to an obviously decreased OS, whereas ATB use later in the disease course did not seem to alter OS in NSCLC patients treated with ICIs [189]. For patients with muscle-invasive bladder cancer, a retrospective analysis found that concomitant ATB therapy with neoadjuvant pembrolizumab was correlated with lower rates of complete response and shorter recurrence-free survival [190]. In patients with a urothelial carcinoma, ATB use was associated with worse survival in patients treated with atezolizumab, whereas no association was observed in patients treated with chemotherapy [191]. A multivariate analysis highlighted that cumulative (multiple or prolonged) courses were an independent negative predictor of survival in patients with advanced cancer receiving ICIs [192]. These results indicate that the gut microbiome and ATB use may be potential biomarkers in predicting the response to immunotherapy. Manipulating the gut microbiome may modulate antitumor activity and improve immune checkpoint blockade efficacy.

### 4.3. Viral Infection

Viral infection is associated with tumorigenesis in a subset of cancers, like Epstein–Barr virus (EBV) in nasopharyngeal cancer and gastric cancer, human papillomavirus (HPV) in head and neck squamous cell carcinoma and cervical cancer, and Merkel cell polyomavirus (MCPyV) in Merkel cell carcinoma. EBV+ gastric cancers have robust PD-L1 expression in both tumor cells and TILs that is not seen in other gastric cancers, suggesting that EBV+ gastric cancer may have a greater likelihood to respond to a PD-1/PD-L1 blockade [193]. Notably, EBV+ gastric cancers have been found to exhibit high response rates to pembrolizumab regardless of mutational load status [136]. The phase II POLARIS-02 trial has recently shown that an early decrease in EBV DNA copy number was associated with a favorable response to toripalimab in subsequent-line treatment of nasopharyngeal carcinomas [194]. In head and neck squamous cell carcinoma, HPV+ status correlated with a T-cell-inflamed gene expression profile and increased immune cytolytic activity, indicating that HPV status could be useful in predicting the effectiveness of PD-1/PD-L1 inhibitors. A further meta-analysis demonstrated that HPV+ patients achieved superior OS than HPV- patients with anti-PD-1/PD-L1 therapy [137]. A multicenter phase II trial assessed first-line therapy with pembrolizumab in patients with advanced Merkel cell carcinoma. They found that PD-L1 expression was more frequent in MCPyV+ tumors than in MCPyV- tumors, whereas responses were observed in both virus-positive tumors and virus-negative tumors [30]. Taken together, viral infection can be evaluated as a potential predictor of PD-1/PD-L1 inhibitors; however, the correlation between viral infection and treatment efficacy needs to be confirmed by additional basic research and large-scale clinical trials.

### 4.4. Corticosteroid Use

Corticosteroids are frequently administered to cancer patients due to their anti-inflammatory, immunosuppressing, and other important pharmacological effects. Given the immunosuppressive effects of corticosteroids, many studies have investigated their impact on the efficacy of a PD-1/PD-L1 blockade. It has been shown that baseline corticosteroid use of the equivalent of ≥10 mg of prednisone was correlated with inferior clinical outcomes in NSCLC patients who were treated with a PD-1/PD-L1 blockade [135]. A retrospective analysis demonstrated that concurrent dexamethasone use limited the clinical benefit of ICIs in glioblastoma [195]. Another retrospective study also showed that high-dose prednisone at time of immunotherapy initiation limited the clinical benefit of an immune checkpoint blockade. However, there was no significant difference in patients receiving ≥10 mg of prednisone for cancer-unrelated indications compared with patients receiving 0 to <10 mg of prednisone, suggesting that the negative effect of high-dose prednisone was likely confounded by the poor-prognosis of the subgroup of patients receiving corticosteroids for palliative indications [196]. Additional prospective studies are warranted to identify if the administration of standard corticosteroids has an impact on ICI efficacy.

### 4.5. Previous Treatment and Response

Increasing amounts of data have shown that radiotherapy stimulates a systemic immune response by enhancing the diversity and presentation of tumor-associated antigens. Therefore, there is a possibility that patients previously treated with radiotherapy may experience enhanced antitumor activity from an immune checkpoint blockade. It has been demonstrated that radiotherapy can trigger the regression of metastatic lesions that were distant from the primary site of irradiation, a phenomenon that is referred to as the abscopal effect. Thus, it is possible that the combination of immunotherapy and radiotherapy can amplify the antitumor immune response, which in turn, is more likely to induce an abscopal effect [197]. A secondary analysis of the KEYNOTE-001 trial [138] investigated the impact of previous radiotherapy on the activity of pembrolizumab in NSCLC. The study showed that both PFS and OS were longer in patients who previously received radiotherapy compared to those without history of previous radiotherapy. In two other studies of NSCLC patients, no significant difference in outcomes was noted between those who received prior radiotherapy and those without previous radiotherapy [198,199]. The benefit of adding concurrent radiotherapy to immunotherapy remains unclear.

NSCLC patients who responded to the treatment immediately before nivolumab (either chemotherapy, curative-intent chemoradiotherapy, or palliative radiotherapy) showed a higher ORR than the non-responders. A subgroup analysis revealed that responders to palliative radiotherapy had better ORR compared to non-responders [139]. A previous response to immunotherapy also offers valuable prognostic information for current checkpoint blockade therapy. Patients with PFS ≥ 90 days after receiving ipilimumab had better clinical benefit with subsequent pembrolizumab compared to patients with PFS < 90 days, although there was no significant difference in ORR between the two groups. Thus, previous treatment history and response status may also provide predictive information for checkpoint inhibitor immunotherapy; however, these findings need to be further validated in larger studies.

### 4.6. Immune-Related Adverse Events

The mechanism of ICI immunotherapy is based on allowing a hindered immune system to recognize and eliminate tumor cells. However, these actions lead to increased risks of immune-related adverse events (i.e., irAEs) [200]. Multiple studies have shown that irAEs are associated with better prognosis and responses to immunotherapy. For example, the occurrence of vitiligo was associated with improved ORR in a melanoma cohort treated with pembrolizumab [142]. Vitiligo and rash were also demonstrated to be correlated with improved OS in melanoma patients treated with nivolumab [144,201]. In non-melanoma patients treated with nivolumab or pembrolizumab, there was also a significant association between low-grade irAEs and improved ORR [143]. A prospective cohort study [141] investigated the association between early irAEs and outcome in NSCLC patients treated with nivolumab and reported that ORR and disease control rates were higher in patients with common irAEs (rash, pyrexia, and diarrhea) than those without irAEs. However, a recent study showed no significant association between irAEs and pembrolizumab efficacy [202]. Thus, because the sample sizes in these studies are small and some studies are too underpowered to detect significant differences, further prospective evaluation with larger cohorts is warranted.

## 5. Challenges and Perspectives

Despite encouraging results from preclinical and clinical studies, there are still many challenges in identifying reliable biomarkers for anti-PD-1/PD-L1 therapy. One challenge is the lack of a uniform standard for currently accessible biomarkers. For example, different antibodies and platforms are recommended for the detection of PD-L1. The thresholds and scoring systems for PD-L1 positivity are highly variable across studies. None of the current biomarkers have been reported consistently and definitively in predicting the efficacy of immunotherapy. Moreover, most of the biomarkers are currently broad spectrum, and further studies are needed to clarify whether any of proposed biomarkers are more specific for a particular ICI to guide better clinical practice.

The second challenge is the complex interactions between tumor, TME, and host immunity. The interaction between tumor cells and TME could lead to remarkable changes in the immune response. Thus, it is necessary to make a comprehensive assessment that includes the immune microenvironment in order to predict the response to treatment. Advanced technologies such as next-generation sequencing and single-cell RNA sequencing can provide a high-resolution of TME.

The third challenge is the limited prediction efficacy of any single biomarker. More and more studies have shown that a single biomarker is not reliable in predicting the efficacy of ICIs. Thus, a combination of biomarkers can be an optimal solution to improve the predictive performance to ICI response. In addition to anti-PD-1/PD-L1 monotherapy, combination immunotherapy and chemotherapy can also lead to promising efficacies in cancer treatment [152]. This highlights the importance of combined biomarkers to predict the response to chemo-immunotherapy.

Another major issue that should be addressed is the ITH, which represents a dynamic alteration of biomarkers during therapy. It is not sufficiently precise to make a clinical decision based on a single sampling from one tumor site or one time point. We need close monitoring of biomarkers since they are dynamic and can evolve during immunotherapy. However, sometimes it is not feasible to obtain tissue samples during ICI treatments. Thus, liquid biopsies have been presented as a promising strategy and can provide peripheral blood markers such as ctDNA and circulating tumor cells [203,204]. These novel techniques enable a complete assessment of the genomic landscape during cancer treatment. To ensure clinical utility and reproducibility, it is crucial to establish a consensus on the methodological standardization for assessing these candidate biomarkers and investigate them in larger patient populations.

## 6. Conclusions

The emergence of anti-PD-1/PD-L1 monoclonal antibodies in cancer therapy holds great promise in improving the clinical outcomes of patients with various cancer types. Identifying reliable predictive biomarkers is of critical importance. PD-L1 expression, MSI, and TMB are by far the best studied candidate biomarkers, yet their value remains controversial. Host–tumor interactions and TME are also associated with the response to ICIs. Multi-parameter models would likely be more effective than single-parameter indicators, but further investigation and validation are warranted. Future biomarker-driven prospective studies are needed to confirm the predictive value of promising biomarkers in immunotherapy, as well as to establish these powerful biomarkers for application into routine clinical practice.
